# “Active Team” a social and gamified app-based physical activity intervention: randomised controlled trial study protocol

**DOI:** 10.1186/s12889-017-4882-7

**Published:** 2017-11-02

**Authors:** Sarah Edney, Ronald Plotnikoff, Corneel Vandelanotte, Tim Olds, Ilse De Bourdeaudhuij, Jillian Ryan, Carol Maher

**Affiliations:** 1 Alliance for Research in Exercise, Nutrition, and Activity, University of South Australia, GPO Box 2471, Adelaide, South Australia Australia; 2 Priority Research Centre for Physical Activity and Nutrition, Advance Technology Centre, Level 3, The University of Newcastle, Newcastle, Australia; 3 Physical Activity Research Group, School of Human Health and Social Sciences, Central Queensland University, Rockhampton, QLD Australia; 4 Department of Movement and Sports Sciences, University of Ghent, Ghent, Belgium

**Keywords:** Social network, Gamification, Behaviour change, Intervention, Social media, Physical activity, Mobile, mHealth, eHealth, Facebook

## Abstract

**Background:**

Physical inactivity is a leading preventable cause of chronic disease and premature death globally, yet over half of the adult Australian population is inactive. To address this, web-based physical activity interventions, which have the potential to reach large numbers of users at low costs, have received considerable attention. To fully realise the potential of such interventions, there is a need to further increase their appeal to boost engagement and retention, and sustain intervention effects over longer periods of time. This randomised controlled trial aims to evaluate the efficacy of a gamified physical activity intervention that connects users to each other via Facebook and is delivered via a mobile app.

**Methods:**

The study is a three-group, cluster-RCT. Four hundred and forty (440) inactive Australian adults who use Facebook at least weekly will be recruited in clusters of three to eight existing Facebook friends. Participant clusters will be randomly allocated to one of three conditions: (1) waitlist control condition, (2) basic experimental condition (pedometer plus basic app with no social and gamification features), or (3) socially-enhanced experimental condition (pedometer plus app with social and gamification features). Participants will undertake assessments at baseline, three and nine months. The primary outcome is change in total daily minutes of moderate-to-vigorous physical activity at three months measured objectively using GENEActive accelerometers [Activeinsights Ltd., UK]. Secondary outcomes include self-reported physical activity, depression and anxiety, wellbeing, quality of life, social-cognitive theory constructs and app usage and engagement.

**Discussion:**

The current study will incorporate novel social and gamification elements in order to examine whether the inclusion of these components increases the efficacy of app-based physical activity interventions. The findings will be used to guide the development and increase the effectiveness of future health behaviour interventions.

**Trial registration:**

This trial was registered with the Australian and New Zealand Clinical Trial Registry (ACTRN12617000113358, date of registration 23 January, 2017).

## Background

Physical inactivity is the fourth leading behavioural risk factor contributing to the population attributable burden of disease, as it is strongly associated with physical and mental health problems and continues to have a significant economic burden [1–4]. Current Australian guidelines recommend adults complete at least 150 min of physical activity per week at a moderate-to-vigorous intensity to reduce the risk of developing chronic diseases such as cardiovascular diseases and diabetes [2, 5, 6], and for a myriad of additional benefits such as improved muscular and cardiorespiratory fitness, stronger bones and mental health [5, 7]. Yet almost half of the Australian adult population do not meet these guidelines [2, 8].

Smartphone and social media use have become ingrained into everyday life and together offer a feasible, accessible and innovative platform from which to launch a physical activity intervention [9–12]. Facebook attracts 15 million Australian users each month and 10 million every day [13], 90% of whom log on from an application (‘app’) on their mobile device [14, 15]. Health behaviours have been found to spread through individuals’ social networks, by way of real life social support [16–18] and this may be true for online social networks [19–23]. Two recent systematic reviews suggest that online social networks may successfully be harnessed to promote health behaviour change [9, 24]. Further exploration is warranted as to date such interventions typically report low rates of participant engagement with intervention components and low rates of retention, potentially limiting their effectiveness [9, 24, 25]. Participant engagement has been positively associated with both increased retention and intervention effects [24, 26–30]. Novel approaches to increase the appeal of, and therefore engagement with, interventions are needed. Gamification, the application of game design elements such as rewards, challenges and competition, teamwork, point scoring and leader boards, into a traditionally non-game environment is one such approach [31, 32]. A recent systematic review suggests that gamification is effective in increasing engagement with online programs [33] and two reviews of commercially available health and fitness apps suggested that gamification has the potential to enhance positive behaviour change [34, 35]. Recent health behaviour change studies incorporating gamification elements have produced similarly promising results [27, 36, 37].

App-based physical activity interventions that incorporate gamification have the potential to reach large numbers of users at relatively low costs [38]. The available evidence highlights a need to enhance user engagement to realise the potential impact of this approach. As such, the current study will assess the efficacy of an app-based intervention that incorporates social media and gamification elements in a three-group cluster-randomised controlled trial (RCT).

### Objectives

The primary aim of this study is to examine whether using the ‘Active Team’ smartphone app, a purpose-built, gamified physical activity intervention that connects users to each other via Facebook, leads to a significant difference in physical activity levels relative to a basic app-based experimental condition and a waitlist control condition after three months.

Secondary aims are to examine whether: (1) differences in physical activity levels between experimental conditions and waitlist control condition are sustained across a nine-month follow up, (2) there are differences between the experimental conditions and waitlist control condition in changes to quality of life, depression and anxiety, and wellbeing after three and nine months, (3) to examine potential moderating effects of sociodemographic variables of increasing physical activity, (4) to examine engagement with the Active Team app, and (5) to examine social-cognitive constructs as potential mediators of the intervention efficacy in increasing physical activity levels.

## Methods

### Study design

The study is a parallel, three-group cluster-RCT with assessments at baseline, three and nine months. Ethical approval for the study was obtained from the Human Research Ethics Committee of the University of South Australia and the trial is registered with the Australian and New Zealand Clinical Trial Registry, protocol number: ACTRN12617000113358. The study is funded by a Project Grant from the National Health and Medical Research Council of Australia. The conduct and reporting of the trial will adhere to the Consolidated Standards of Reporting Trials (CONSORT) guidelines [39]. All participants will provide informed consent online prior to commencing the study.

### Participants

#### Eligibility criteria

Participants will be aged 18 to 65 years, use Facebook at least weekly, be fluent in English, live anywhere in Australia, currently complete less than 150 min of moderate-to-vigorous physical activity (MVPA) per week and be able to assemble a cluster of minimum three to maximum eight Facebook friends who also meet the eligibility criteria and are willing to join the study. Participants who self-report as being unable to safely increase their physical activity levels will be excluded from the study.

#### Recruitment

Four hundred and forty (440) participants will be recruited over a period of nine months. Recruitment strategies include flyers placed around local university campuses, paid Facebook advertising (including a campaign targeted specifically to men, as previous studies have had difficulties recruiting men for this type of intervention [9]), and free advertisements placed on community group Facebook pages. All advertisements will direct participants to a website containing further study details including full eligibility criteria and a form to register interest.

#### Procedure

**Fig. 1:**
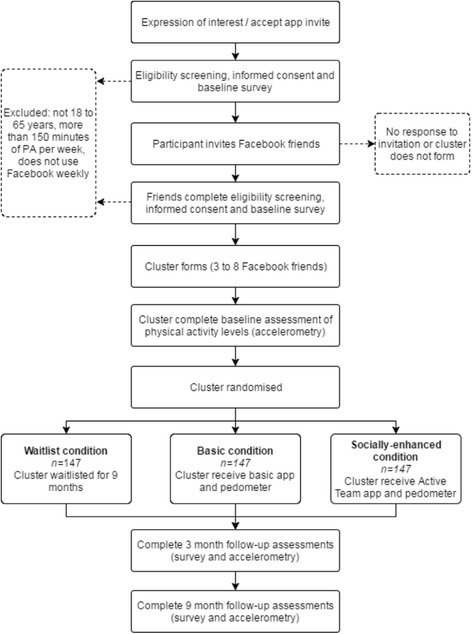
Study Procedure Flowchart

Figure 1 provides an overview of the study procedure.

All participants who register interest will be contacted via email, and followed up via phone. Participants will be asked to complete an online eligibility screening and informed consent process that is embedded within the baseline survey. Participants who meet the eligibility criteria and provide informed consent will be directed to the full baseline survey, which takes approximately 15 min to complete.

As the intervention seeks to use existing social networks to promote physical activity, the study will recruit clusters of between three to eight participants who are already friends on Facebook. Initial participants will be guided through the process of downloading the Active Team app and using it to send invitations to join the study to their selected friends. The invitation will appear as a notification on the recipients’ Facebook profile and those who click on the notification will be automatically directed to their respective app store. New participants joining the study in this way will register their interest via the app and will also be contacted via email, and followed up via phone.

Upon completion of the baseline survey and assembly into a cluster, a wrist-worn accelerometer (GENEActiv) will be posted to each participant in the cluster with instructions to wear the device 24 h a day for seven days before posting it back in a reply-paid envelope. Once all members of a cluster have completed baseline assessments (survey and accelerometry) the cluster will be randomly allocated to either a waitlist control condition, basic experimental condition or socially-enhanced experimental condition on a 1:1:1 allocation ratio using permuted blocks with block sizes of 9, 12 or 15, to ensure a balance of clusters across experimental conditions throughout the trial. An independent allocation officer will determine block size and allocation schedule using a random number generator and a computer generated randomisation schedule. Block size and block allocation sequence will be concealed from the person enrolling the participants.

The assessments procedure will be repeated at three and nine months. Participants will be provided with a small honorarium ($AUD75, about $US55) upon completion of assessments at the final time point.

#### Sample size and statistical power

Sample size calculations are based on the primary outcome measure of total daily minutes of MVPA measured using accelerometry. The pilot study (*n =* 110) of the Active Team app [11] found a small to medium effect size difference (Cohen’s d = 0.39) relative to a waitlist control condition from baseline to eight week follow up. For the current study, a similar effect size difference is anticipated between the socially-enhanced experimental condition and the waitlist control condition. A smaller effect size difference is anticipated between the socially-enhanced experimental condition and the basic experimental condition, hence the larger sample size. Based on sample size calculations (α = 0.05, β = 0.2), a sample of 440 will be sufficient to detect a small effect size (Cohen’s f^2^ = 0.13, Cohen’s d = 0.25) for between-group difference on the primary outcome if one exists.

### Intervention

Active Team is a mobile app designed in conjunction with a software development company (Portal Australia) to encourage inactive adults to meet the current guidelines of engaging in a minimum of 150 min of moderate or vigorous intensity physical activity per week by encouraging participants to take 10,000 steps per day. An earlier version of the Active Team software delivered as a Facebook app has been pilot tested [11] and subsequently undergone redevelopment and improvement for release as an iPhone and Android app.

Social-cognitive theory (SCT), in particular the need to increase self-efficacy as a key driver of behaviour adoption and maintenance [40, 41], guided the development of the Active Team intervention and the inclusion of app features. The social and gamification features are inherently linked with one another, and are designed to mimic real-life social interactions and to capitalise on the social comparison, support and influence found within existing online friendship groups, in order to motivate health behaviour change [42]. The app prompts self-monitoring of behaviour and mastery experience by achieving a daily goal [43]. Taken together, these features are intended to aid participant retention by increasing the appeal of the app and therefore the likelihood that a participant will choose to keep returning to the app and engaging with the features.

**Fig. 2:**
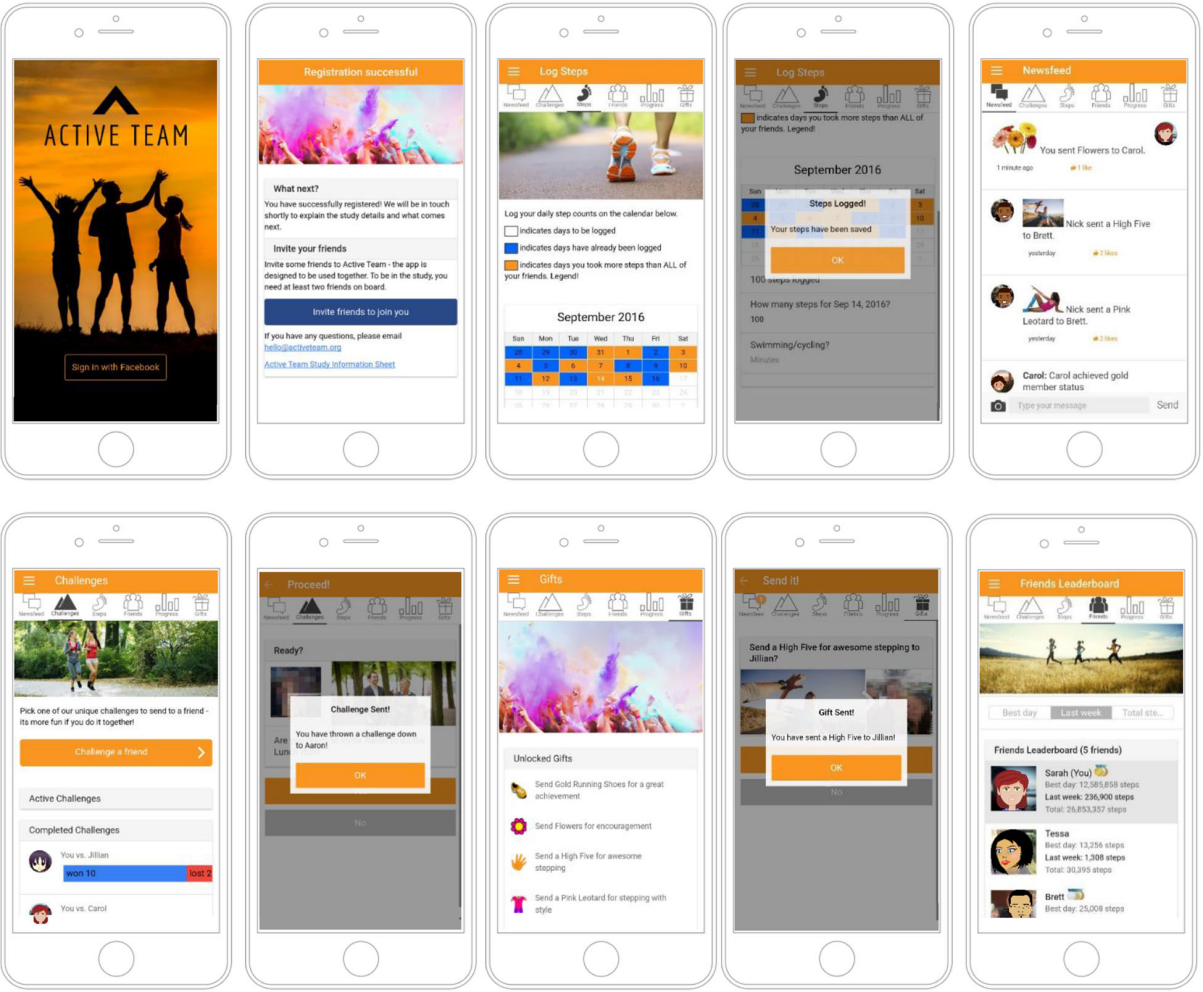
Screenshots of the Active Team app showing (left to right, clockwise): splashscreen, registration, step-logging calendar, step logging confirmation, newsfeed, challenges, and challenge sent confirmation, gifts, confirmation of gift sent, and leader board

During the recruitment process, all participants will see the registration screens of the app, and following randomisation, participants will only be given access to the features relevant to their allocated experimental condition. To prevent contamination between experimental conditions, waitlist control and basic experimental condition participants will be blocked from receiving notifications from Facebook friends in the socially-enhanced experimental condition, if they have such friends. Screenshots of the Active Team app are shown in Fig. 2.

#### Waitlist control condition

Participants allocated to the waitlist control condition will not be able to progress past the registration screen of the app until the end of the nine-month study period. As such, they have no access to any of the app features that may help them in becoming more physically active, and will be encouraged to continue with their usual activities.

#### Basic experimental condition

Participants in the basic experimental condition will receive a pedometer and access to the app’s self-monitoring features but no social and gamification functions.

Daily steps will be counted using a wrist-worn pedometer (Zencro, TW64S) which the participant then manually enters into a calendar in the app. The app tracks the days where steps have been logged and provides a summary of progress. Participants will receive a daily ‘push notification’ (an alert that automatically pops up on their mobile device) with a reminder to log their daily step count, and the timing of the daily reminder can be customised within the app’s settings. Participants will also receive a weekly email which provides a summary of their step count progress and encouragement to use the app. Participants are able to opt out of receiving the push notification and the weekly email, if they choose to do so.

#### Socially-enhanced experimental condition

Participants in the socially-enhanced experimental condition will receive a wrist-worn pedometer (Zencro, TW64S) and have access to the full Active Team app with the same self-monitoring capabilities and weekly email reminder as those in the basic experimental condition plus the addition of push notifications and social and gamification features.

The socially-enhanced experimental condition app links to Facebook and allows users to interact with their Facebook friends who are also using the app and are assigned to the same condition. Within the app, participants are able to post messages and photographs on a Facebook-style newsfeed, to send and receive virtual gifts, to compete against one another for the highest daily and weekly step count on the leader board, and to compete in mini-challenges that encourage short bursts of physical activity. Examples of the mini-challenges include the ‘Step Sprinter’ challenge to complete 2000 steps in the next 20 mins, and the ‘Three Day Step Streaker’ challenge where participants take at least 12,000 steps per day, for three consecutive days. Participants in the socially-enhanced experimental condition receive a daily ‘push notification’ with a reminder to log their daily step count, as well as push notifications when one of their friends has interacted with them in the app. Participants will also receive a weekly email which provides a summary of their step count progress and encouragement to use the app. The timing of the daily push notification can be customised within the app’s settings, and participants are able to opt out of receiving the push notifications and the weekly email, if they choose to do so.

### Outcomes

Participant demographic information including residential address, date of birth, gender, marital status, height and weight status, country of birth, highest education level and details of employment status, will only be collected in the baseline survey.

#### Primary outcome

The primary outcome measure is objective daily minutes of MVPA measured using GENEActiv accelerometers [Activinsights Ltd., UK] at baseline, three and nine months [44]. GENEActiv accelerometers are small devices worn on the wrist that measure the frequency, duration and intensity of physical activity in real time. Participants will wear an accelerometer on their left wrist 24 h a day for seven days at each time-point, with the exception of during water-based activities. When the accelerometer is returned the data will be inspected to ensure the minimum compliance threshold of at least 10 h of wear whilst awake, on at least four days including a minimum of one weekend day is met [45]. Participants returning incomplete data will be asked to wear the accelerometer again, up to a total of three times. For baseline assessments, participants who decline to wear the accelerometer again or who return incomplete data three times will be excluded from the study.

The reliability and validity of GENEActiv accelerometers and secondary outcome instruments are presented in Table 1.

**Table 1:** Description, reliability and validity of outcome measures

Construct	Instrument	Description	Reliability	Validity
Objective physical activity	GENEActiv Accelerometer	Wrist-worn accelerometers to measure frequency, duration and intensity of physical activity in real-time	Excellent overall intra- and inter- instrument reliability for activity count against a mechanical shaker (CVintra =1.4%, CVinter =2.1%) [59].	*r =* 0.89 (95% CI, 0.84–0.94) when compared to indirect calorimetry [59].
Self-reported physical activity	Active Australia Survey (AAS) [46].	Self-report, 8-items, to measure frequency, duration, intensity and information about the type and context of physical activity	Test-retest reliability (24 h apart) of 0.52 (95% CI, 0.44–0.60 [60].	Moderate correlation (*r =* 0.49–0.64, *p* < 0.01) when compared to objective accelerometry data [61].
Quality of life	Short Form 12-Item Health Survey (SF-12) [48].	Self-report, 12-items, health-related quality of life (mental and physical health)	Test-retest reliability (two weeks apart): Physical Component Summary 0.89, and Mental Component Summary 0.76 [48].	SF-12 PCS and MCS scores are correlated with PCS-36 (*r* = 0.951) and MCS-36 (*r* = 0.969) and equates to an *R* ^2^ of 0.904 and 0.939, respectively [48]. SF-36 PCS, MCS and global scores are correlated with SIP Physical (*r =* 0.67), SIP Psychological (*r =* 0.70) and SIP Global (*r =* 0.78) [62].
Emotional state	Depression Anxiety Stress Scale (DASS-21) [50].	Self-report, 21-items, negative emotional states related to depression, anxiety and stress over the past week	Test-retest reliability (three weeks apart) 0.77 (95% CI, 0.56–0.88), 0.89 (95% CI, 0.81–0.94), and 0.85 (95% CI, 0.51–0.94) for Depression, Anxiety and Stress, respectively [63].	Correlation coefficients of 0.79 for depression scale when compared to BDI, 0.85 for anxiety scale when compared to BAI, and 0.68 for stress scale when compared to STAI-T [64].
Psychological well-being	PERMA-Profiler Measure [54].	Self-report, 23-items, well-being and happiness in relation to the five components identified by Seligman: emotion, engagement, relationships, meaning and accomplishment [55].	Test-retest reliability (two weeks apart) 0.84 for positive emotions, 0.78 for engagement, 0.83 for relationships, 0.86 for meaning and 0.80 for accomplishment [54].	Correlation coefficients of 0.79 when compared to SWLS, 0.81 when compared to WEMWBS and 0.87 when compared to FS [54].

*PCS* Physical Composite Scale, *MCS* Mental Composite Scale, *SIP* Sickness Impact Profile, *BDI* Beck Depression Inventory, *BAI* Beck Anxiety Inventory, *STAI-T* State-Trait Anxiety Inventory – Trait Version, *SWLS* Satisfaction with Life Scale, *WEMWBS* Short Warwick-Edinburgh Mental Well-being Scale, *FS* Flourishing Scale

#### Secondary outcomes

Self-reported physical activity will be collected to supplement the objective physical activity data from accelerometry, as it provides information about the type and context of physical activity. These data will be collected using the Active Australia Survey (AAS) [46], an 8-item survey that asks participants to recall leisure-based physical activity levels within the previous week (e.g., brisk walking, water-based activities, moderate-to-vigorous leisure activities, and vigorous household chores) including frequency, duration and intensity of activity. For example, ‘In the last week, how many times have you walked continuously, for at least 10 minutes, for recreation, exercise, or to get to or from places?’ and, ‘In the last week, how many times did you do any other more moderate physical activities that you have not already mentioned?’.

Sleep quality and quantity will be assessed using 3-items adapted from the validated Pittsburgh Sleep Quality Index [47]. Participants will be asked to report their usual sleep and wake time for the past month, and to rate their overall sleep quality on a 4-point scale (0 = very good, 1 = good, 2 = bad, 3 = very bad).

Health-related quality of life will be measured using the SF-12 Health Survey [48]. The validated SF-12 is a condensed version of the original SF-36, and is suitable for use with large sample sizes. The SF-12 assesses quality of life in relation to self-reported physical and mental health outcomes [49]. Questions relate to whether physical or mental health has prohibited or had an adverse effect on engagement in daily life and incidental physical activities (e.g. household chores, social activities, work, taking a flight of stairs) over the past four weeks, whether the participant has experienced pain that has interfered with their life, and the participants’ emotions experienced over the past four weeks (e.g., sad and blue, energy levels).

The Depression and Anxiety Stress Scale (DASS-21) is a 21-item self-assessment of the severity of symptoms relating to depression and anxiety, including any changes over time and is not intended to be used as a diagnostic tool [50]. Respondents are asked to consider each of the 21 items in the context of their previous week and answers are scored on a 4-point Likert scale. For example: ‘I found it difficult to relax’ (0 = never, 1 = sometimes, 2 = often, 3 = almost always), ‘I was intolerant of anything that kept me from getting on with what I was doing’ (0 = never, 1 = sometimes, 2 = often, 3 = almost always), and ‘I felt scared without any good reason’ (0 = never, 1 = sometimes, 2 = often, 3 = almost always).

Social-cognitive theory constructs (21 items) will measure self-efficacy, expectations, barriers [51], intentions [52] and goals [53] related to performance of physical activity. For example: ‘I am motivated to get at least 30 minutes of physical activity on five or more days per week’ (1 = not at all motivated, 2 = not very motivated, 3 = neutral, 4 = very motivated, 5 = extremely motivated’), and ‘I am confident that I can get at least 30 minutes of physical activity when I have many other demands on my time’ (1 = not at all confident, 2 = not very confident, 3 = moderately confident, 4 = very confident, 5 = extremely confident).

The PERMA Profiler Measure [54] will be used to assess self-reported psychological well-being in relation to the five elements of happiness identified by Seligman [55]: positive emotion, engagement, relationships, meaning and accomplishments. For example: ‘In general, how often do you feel joyful?’ (0 = never, 10 = always), ‘To what extent do you receive help and support from others when you need it?’ (0 = not at all, 10 = always), ‘In general, to what extent do you lead a purposeful and meaningful life?’ (0 = not at all, 10 = completely), and ‘How often do you achieve the important goals you have set for yourself?’ (0 = never, 10 = always).

Engagement with the app will be assessed using app usage data that will automatically upload to a secure server in real-time as participants are using the app, and the usability of the Active Team app will be assessed using the System Usability Scale (10 items) [56] and further purpose-designed feedback questions (18 items).

#### Statistical analyses

Intention-to-treat analysis [57] and random effects mixed modelling will be used to assess whether there are significant differences in changes to the primary outcome measure (total daily minutes of MVPA measured from GENEActiv accelerometers) between experimental conditions over time (i.e., baseline, three and nine months). The most appropriate procedure for handling missing data will be selected after inspecting the amount and pattern of missingness. Random effects mixed modelling will also be used to explore potential changes in secondary outcomes including quality of life, depression and anxiety, and wellbeing. Planned subgroup analyses include examining any moderating effects of sociodemographic variables and examining social-cognitive constructs and app usage as potential mediators for increases in physical activity levels.

## Discussion

Smartphones and social media have become embedded within everyday life, and offer a promising platform for a physical activity intervention. This study seeks to overcome limitations of previous studies that report low rates of participant engagement and retention and weak short-term effects [11, 24] by capitalising on the social support and influence found within existing friendship networks, and incorporating social and gamification features. Taken together, these features are intended to add to the appeal of the app, in turn increasing user engagement with the intervention to promote retention and sustain behaviour change over time.

Findings from the three-group RCT design, with the inclusion of a waitlist control, basic and socially-enhanced experimental condition, will allow for detailed examination of which intervention components have the greatest potential to increase the efficacy of app-based interventions. In particular, whether the addition of social and gamification features lead to an increase in intervention efficacy. Further strengths of our study include the rigorous, well-planned and pre-specified study protocol that has been reported as per Standard Protocol Items: Recommendations for Interventional Trials (SPIRIT) guidelines [58], the use of an objective measure of physical activity as the primary outcome, rigorous randomisation and allocation concealment procedure, the large and nationally-based sample, inclusion of a nine month follow-up, the cost-effective ‘hands-off’ intervention delivery approach that requires no face-to-face contact, as well as email and push notification strategies that are in place to draw participants back to the intervention.

Active Team is a low-cost and easily accessible health care intervention delivered via technology that is already embedded within everyday life. Findings from the pilot study are promising [11] and further enhancements, in the form of additional social gamification features, have now been added and will now be examined within the context of a large and rigorous RCT. Our findings will contribute to and extend the current evidence of how to mitigate challenges caused by physical inactivity, and with broad potential for use in targeting other health behaviours.

## Abbreviations

AAS: Active Australia Survey
App: Mobile application
CONSORT: Consolidated Standards of Reporting Trials
MVPA: Moderate-to-vigorous physical activity
RCT: Randomised controlled trial
SCT: Social-cognitive constructs
SPIRIT: Standard Protocol Items: Recommendations for Interventional Trials

## References

[1] Australian Institute of Health and Welfare (2011). Key indicators of progress for chronic disease and associated determinants.

[2] Australian Institute of Health and Welfare (2014). Australia’s health 2014.

[3] World Health Organisation (2011). Global status report on noncommunicable diseases 2010: description of the global burden of NCDs, their risk factors and determinants.

[4] Institute of Health Metrics and Evaluation (2013). The global burden of disease: generating evidence, guiding policy.

[5] World Health Organisation (2010). Global recommendations on physical activity for health.

[6] Blair SN, Morris JN (2009). Healthy hearts—and the universal benefits of being physically active: physical activity and health. Ann Epidemiol.

[7] Zschucke E, Gaudlitz K, Ströhle A (2013). Exercise and physical activity in mental disorders: clinical and experimental evidence. J Prev Med Public Health.

[8] Australian Bureau of Statistics (2015). Australian health survey: first results, 2014–15.

[9] Maher CA, Lewis LK, Ferrar K, Marshall S, De Bourdeaudhuij I, Vandelanotte C (2014). Are health behavior change interventions that use online social networks effective? A systematic review. J Med Internet Res.

[10] Maher C, Ryan J, Kernot J, Podsiadly J, Keenihan S (2016). Social media and applications to health behavior. Curr Opin Psychol.

[11] Maher C, Ferguson M, Vandelanotte C, Plotnikoff R, De Bourdeaudhuij I, Thomas S, Nelson-Field K, Olds T (2015). A web-based, social networking physical activity intervention for insufficiently active adults delivered via Facebook app: randomized controlled trial. J Med Internet Res.

[12] Cavallo DN, Tate DF, Ries AV, Brown JD, DeVellis RF, Ammerman AS (2012). A social media–based physical activity intervention: a randomized controlled trial. Am J Lifestyle Med.

[13] Sensis: Social media report May 2015. Sensis. 2015. https://www.sensis.com.au/assets/PDFdirectory/Sensis_Social_Media_Report_2015.pdf. Accessed 24 Mar 2017.

[14] Heber A. These incredible stats show exactly how huge Facebook is in Australia. In: Business Insider Australia. Allure Media. 2015. http://www.businessinsider.com.au/these-incredible-stats-show-exactly-how-huge-facebook-is-in-australia-2015-4. Accessed 24 Mar 2017.

[15] Cowling D. Social media statistics Australia – March 2016. In: Social Media News. 2016. http://www.socialmedianews.com.au/social-media-statistics-australia-march-2016/. Accessed 24 Mar 2017.

[16] Christakis NA, Fowler JH (2008). The collective dynamics of smoking in a large social network. N Engl J Med.

[17] Christakis NA, Fowler JH (2007). The spread of obesity in a large social network over 32 years. N Engl J Med.

[18] Hanson CL, Cannon B, Burton S, Giraud-Carrier C (2013). An exploration of social circles and prescription drug abuse through twitter. J Med Internet Res.

[19] Moorhead SA, Hazlett DE, Harrison L, Carroll JK, Irwin A, Hoving C (2013). A new dimension of health care: systematic review of the uses, benefits, and limitations of social media for health communication. J Med Internet Res.

[20] Chang T, Chopra V, Zhang C, Woolford SJ (2013). The role of social media in online weight management: systematic review. J Med Internet Res.

[21] Zhang J, Brackbill D, Yang S, Centola D (2015). Efficacy and causal mechanism of an online social media intervention to increase physical activity: results of a randomized controlled trial. Prev Med Rep.

[22] Valle CG, Tate DF, Mayer DK, Allicock M, Cai J (2013). A randomized trial of a Facebook-based physical activity intervention for young adult cancer survivors. J Cancer Surviv.

[23] Monroe CM, Bassett DR, Fitzhugh EC, Raynor HA, Thompson DL (2016). Effect of adding online social support tools to an adult walking program: a pilot randomized controlled trial. Health Promot Pract.

[24] Laranjo L, Arguel A, Neves AL, Gallagher AM, Kaplan R, Mortimer N, Mendes GA, Lau AYS (2015). The influence of social networking sites on health behavior change: a systematic review and meta-analysis. J Am Med Inform Assoc.

[25] Davies C, Corry K, Van Itallie A, Vandelanotte C, Caperchione C, Mummery K (2012). Meta-analysis of internet-delivered interventions to increase physical activity levels. Int J Behav Nutr Phys Act.

[26] Turner-McGrievy GM, Tate DF (2013). Weight loss social support in 140 characters or less: use of an online social network in a remotely delivered weight loss intervention. Transl Behav Med..

[27] Leahey T, Rosen J (2014). Dietbet: a web-based program that uses social gaming and financial incentives to promote weight loss. J Med Internet Res.

[28] Cavallo DN, Tate DF, Ward DS, DeVellis RF, Thayer LM, Ammerman AS (2014). Social support for physical activity—role of Facebook with and without structured intervention. Transl Behav Med.

[29] Napolitano MA, Hayes S, Bennett GG, Ives AK, Foster GD (2013). Using Facebook and text messaging to deliver a weight loss program to college students. Obesity.

[30] Rote AE, Klos LA, Brondino MJ, Harley AE, Swartz AM (2015). The efficacy of a walking intervention using social media to increase physical activity: a randomized trial. J Phys Act Health.

[31] Deterding S, Dixon D, Khaled R, Nacke L (2011). From game design elements to gamefulness: defining “gamification”. Proceedings of the 15th international academic MindTrek conference: envisioning future media environments.

[32] Miller AS, Cafazzo JA, Seto E (2016). A game plan: gamification design principles in mhealth applications for chronic disease management. Health Inform J.

[33] Looyestyn J, Kernot J, Boshoff K, Ryan J, Edney S, Maher C (2017). Does gamification increase engagement with online programs? A Systematic Review. PLoS One.

[34] Bardus M, van Beurden SB, Smith JR, Abraham C (2016). A review and content analysis of engagement, functionality, aesthetics, information quality, and change techniques in the most popular commercial apps for weight management. Int J Behav Nutr Phys Act.

[35] Lister C, West JH, Cannon B, Sax T, Brodegard D (2014). Just a fad? Gamification in health and fitness apps. JMIR Serious Games.

[36] Schoech D, Boyas JF, Black BM, Elias-Lambert N (2013). Gamification for behavior change: lessons from developing a social, multiuser, web-tablet based prevention game for youths. J Technol Hum Serv.

[37] Davies C, Corry K, Van Itallie A, Vandelanotte C, Caperchione C, Mummery WK (2012). Prospective associations between intervention components and website engagement in a publicly available physical activity website: the case of 10,000 steps Australia. J Med Internet Res.

[38] Schoeppe S, Alley S, Van Lippevelde W, Bray NA, Williams SL, Duncan MJ, Vandelanotte C (2016). Efficacy of interventions that use apps to improve diet, physical activity and sedentary behaviour: a systematic review. Int J Behav Nutr Phys Act.

[39] Moher D, Hopewell S, Schulz KF, Montori V, Gotzsche PC, Devereaux PJ, Elbourne D, Egger M, Altman DG. CONSORT 2010 explanation and elaboration: updated guidelines for reporting parallel group randomised trials. BMJ 2010; doi:10.1136/bmj.c869. 10.1136/bmj.c869 PMC2844943 20332511

[40] Bandura A. Self-efficacy: the exercise of control. United States: Worth Publishers; 1997.

[41] Bandura A (2004). Health promotion by social cognitive means. Health Educ Behav.

[42] Smith KP, Christakis NA (2008). Social networks and health. Annu Rev Sociol.

[43] Abraham C, Michie S (2008). A taxonomy of behavior change techniques used in interventions. Health Psychol.

[44] Activinsights Ltd, Geneactiv. 2008: Cambridge.

[45] Trost SG, McIver KL, Pate RR (2005). Conducting accelerometer-based activity assessments in field-based research. Med Sci Sports Exerc.

[46] Australian Institute of Health and Welfare (2003). The active Australia survey: a guide and manual for implementation, analysis and reporting.

[47] Buysse DJ, Reynolds CF, Monk TH, Berman SR, Kupfer DJ (1989). The Pittsburgh sleep quality index: a new instrument for psychiatric practice and research. Psychiatry Res.

[48] Ware JE, Kosinski M, Keller SD (1996). A 12-item short-form health survey: construction of scales and preliminary tests of reliability and validity. Med Care.

[49] Wee CC, Davis RB, Hamel MB (2008). Comparing the SF-12 and SF-36 health status questionnaires in patients with and without obesity. Health Qual Life Outcomes.

[50] Lovibond SH, Lovibond PF (1995). Manual for the depression anxiety stress scales.

[51] Plotnikoff RC, Blanchard C, Hotz SB, Rhodes R (2001). Validation of the decisional balance scales in the exercise domain from the transtheoretical model: a longitudinal test. Meas Phys Educ Exerc Sci.

[52] Rhodes RE, Hunt Matheson D, Mark R (2010). Evaluation of social cognitive scaling response options in the physical activity domain. Meas Phys Educ Exerc Sci.

[53] Rovniak LS, Anderson ES, Winett RS, Stephens RS (2002). Social cognitive determinants of physical activity in young adults: a prospective structural equation analysis. Ann Behav Med.

[54] Butler J, Kern ML (2016). The PERMA-profiler: a brief multidimensional measure of flourishing. Int J Wellbeing.

[55] Seligman MEP (2011). Flourish: a visionary new understanding of happiness and well-being.

[56] Brooke J, Jordan PW, McClelland IL, Weerdmeester B (1996). System usability scale - a quick and dirty usability scale. Usability evaluation in industry.

[57] Gupta SK (2011). Intention-to-treat concept: a review. Perspect Clin Res.

[58] Chan AW, Tetzlaff JM, Altman DG, Laupacis A, Gøtzsche PC, Krleža-Jerić K, Hróbjartsson A, Mann H, Dickersin K, Berlin JA, Doré CJ, Parulekar WR, Summerskill WS, Groves T, Schulz KF, Sox HC, Rockhold FW, Rennie D, Moher D (2013). Spirit 2013 statement: defining standard protocol items for clinical trials. Ann Intern Med.

[59] Esliger DW, Rowlands A, Hurst TL, Catt M, Murray P, Eston R (2011). Validation of the GENEA accelerometer. Med Sci Sports Exerc.

[60] Brown WJ, Trost SG, Bauman A, Mummery K, Owen N (2004). Test-retest reliability of four physical activity measures used in population surveys. J Sci Med Sport.

[61] Freene N, Waddington G, Chesworth W, Davey R, Cochrane T (2014). Validating two self-report physical activity measures in middle-aged adults completing a group exercise or home-based physical activity program. J Sci Med Sport.

[62] Katz JN, Larson MG, Phillips CB, Fossel AH, Liang MH (1992). Comparative measurement sensitivity of short and longer health status instruments. Med Care.

[63] Asghari A, Saed F (2008). Psychometric properties of the depression anxiety stress Scales-21 (DASS-21) in a non-clinical Iranian sample. Int J Psychol.

[64] Antony MM, Bieling PJ, Cox BJ, Enns MW, Swinson RP, Haynes SN (1998). Psychometric properties of the 42-item and 21-item versions of the depression anxiety stress scales in clinical groups and a community sample. Psychol Assessment.

